# Characterization of FGF23-Dependent Egr-1 Cistrome in the Mouse Renal Proximal Tubule

**DOI:** 10.1371/journal.pone.0142924

**Published:** 2015-11-20

**Authors:** Anthony A. Portale, Martin Y. H. Zhang, Valentin David, Aline Martin, Yan Jiao, Weikuan Gu, Farzana Perwad

**Affiliations:** 1 Department of Pediatrics, Division of Nephrology, University of California San Francisco, San Francisco, California, United States of America; 2 Department of Medicine, Division of Nephrology/Hypertension, Northwestern University–Feinberg School of Medicine, Chicago, Illinois, United States of America; 3 Department of Orthopaedic Surgery, University of Tennessee Health Science Center, Memphis, Tennessee, United States of America; Nihon University School of Medicine, JAPAN

## Abstract

Fibroblast growth factor 23 (FGF23) is a potent regulator of phosphate (Pi) and vitamin D homeostasis. The transcription factor, early growth response 1 (egr-1), is a biomarker for FGF23-induced activation of the ERK1/2 signaling pathway. We have shown that ERK1/2 signaling blockade suppresses renal egr-1 gene expression and prevents FGF23-induced hypophosphatemia and 1,25-dihydroxyvitamin D (1,25(OH)_2_D) suppression in mice. To test whether egr-1 itself mediates these renal actions of FGF23, we administered FGF23 to *egr-1* ^*-/-*^ and wild-type (WT) mice. In WT mice, FGF23 induced hypophosphatemia and suppressed expression of the renal Na/Pi cotransporters, Npt2a and Npt2c. In FGF23-treated *egr-1* ^*-/-*^ mice, hypophosphatemic response was greatly blunted and Na/Pi cotransporter expression was not suppressed. In contrast, FGF23 induced equivalent suppression of serum 1,25(OH)_2_D concentrations by suppressing renal *cyp27b1* and stimulating c*yp24a1* mRNA expression in both groups of mice. Thus, downstream of receptor binding and ERK1/2 signaling, we can distinguish the effector pathway that mediates FGF23-dependent inhibition of Pi transport from the pathway that mediates inhibition of 1,25(OH)_2_D synthesis in the kidney. Furthermore, we demonstrate that the hypophosphatemic effect of FGF23 is significantly blunted in *Hyp*/*egr-1* ^*-/-*^ mice; specifically, serum Pi concentrations and renal Npt2a and Npt2c mRNA expression are significantly higher in *Hyp*/*egr-1* ^*-/-*^ mice than in *Hyp* mice. We then characterized the egr-1 cistrome in the kidney using ChIP-sequencing and demonstrate recruitment of egr-1 to regulatory DNA elements in proximity to several genes involved in Pi transport. Thus, our data demonstrate that the effect of FGF23 on Pi homeostasis is mediated, at least in part, by activation of egr-1.

## Introduction

Fibroblast growth factor 23 (FGF23) is a bone-derived circulating hormone that is critical for phosphate (Pi) and vitamin D homeostasis. Excess circulating FGF-23 is responsible for the pathogenesis of hypophosphatemic syndromes; specifically, X-linked hypophosphatemia (XLH), autosomal recessive and dominant hypophosphatemic rickets, and tumor induced osteomalacia [1–3]. FGF-23 excess induces renal Pi wasting with resultant hypophosphatemia, 1,25 dihydroxyvitamin D (1,25(OH)_2_D) deficiency, rickets and osteomalacia. The kidney is the primary physiologic target for FGF23 action. FGF23 decreases serum Pi concentrations by directly inhibiting renal Pi reabsorption via the sodium-dependent Pi cotransporters, Npt2a and Npt2c in the renal proximal tubule [4–8]. FGF23 suppresses renal 1,25(OH)_2_D synthesis by directly suppressing *cyp27b1* and stimulating *cyp24a1* gene expression [6,7]; *cyp27b1* and *cyp24a1* encode for the enzymes responsible for the synthesis and degradation of 1,25(OH)_2_D, respectively. In a mouse model of XLH (*Hyp* mouse), we showed that serum FGF23 concentrations are 17-fold higher than in normal mice [7] and mitogen activated protein kinase (MAPK)/extracellular signal regulated kinase 1/2 (ERK1/2) signaling is constitutively activated in the kidney. The transcription factor, early growth response 1 (egr-1), is downstream of ERK1/2 and is a biomarker for activation of ERK1/2 signaling by FGF23 [4,9]. Egr-1 mRNA expression is two-fold higher in *Hyp* mice than in normal mice [10,11]. Blockade of ERK1/2 signaling in the kidney in *Hyp* mice is associated with reduction in egr-1 gene expression and improvements in the hypophosphatemia, 1,25(OH)_2_D deficiency and skeletal mineralization defects [10,11]. Thus, our studies demonstrated that activation of the ERK1/2 signaling pathway by FGF23 is critical to the pathogenesis of hypophosphatemia and 1,25(OH)_2_D deficiency in *Hyp* mice.

The role of egr-1 in the kidney is not well studied, and whether it directly mediates FGF23-dependent regulation of renal Pi and vitamin D metabolism is unknown. To test whether egr-1 itself mediates the renal actions of FGF23, we determined the effects of FGF23 on renal Pi transport and 1,25(OH)_2_D synthesis in egr-1 null (*egr-1* ^*-/-*^) and *Hyp*/*egr-1* ^*-/-*^ mice. To obtain a comprehensive view of the genes regulated by egr-1 in the kidney, we employed chromatin-immunoprecipitation (ChIP) coupled with genome-wide sequencing (ChIP-seq) analysis to characterize the FGF23-dependent egr-1 cistrome in the kidney. We integrated the ChIP-seq data with gene expression profiling data to identify direct targets that are both bound by egr-1 and regulated by the FGF23/egr-1 signaling axis.

## Methods

### Animals

We purchased *egr-1* ^*-/-*^ mice and their wild-type littermates, 50–76 days of age, from Jackson Laboratory (Bar Harbor, ME). Phenotypic features of mice with global egr-1 gene deletion have been described [12]. All mice were fed a constant diet containing 0.6% phosphorus and 1% calcium (Teklad diet 98243, Harlan Laboratories, Madison, WI) starting 4 days before the experiment. Mice were injected intraperitoneally (IP) with vehicle or human recombinant FGF-23 (Genzyme, MA) (150ng/g body weight Q12 x 3 doses) and sacrificed 1 hour after the 3^rd^ dose (n = 8–10 mice per group). Female *Hyp* mice purchased from Jackson Laboratories were crossed with *egr-1* ^*+/-*^ heterozygous males to generate *Hyp*/*egr-1* ^*-/-*^ mice that were studied at 50–80 days of age. For ChIP-seq and microarray experiments, 6 week old C57/Bl6J wild-type mice were administered a single IP dose of FGF23 (150ng/g body weight) or vehicle and sacrificed after 1 and 2 hrs. Blood was obtained for determination of serum biochemistries. The kidneys were removed and frozen for subsequent preparation of RNA and protein samples. All procedures used in this study were approved by the Committee on Animal Research, University of California San Francisco.

### Serum Biochemistry

Serum phosphorus and calcium concentrations were determined using kits from Stanbio Laboratories (San Antonio, TX). Serum 1,25(OH)_2_D concentrations were determined using an enzyme immunoassay (EIA) kit from Immunodiagnostic Systems, Inc. (Scottsdale, AZ). Serum intact parathyroid hormone (PTH) concentrations were determined using EIA kits from Immutopics International (San Clemente, CA).

### Real-Time PCR

Total RNA was isolated from kidney using TRIzol reagent (Invitrogen). Probes and primer sets were custom designed as previously described [11]. The mRNA abundance of the gene of interest, expressed relative to that of gus mRNA, was quantitated by real-time PCR using the ABI 7900 HT Sequence Detection System (Applied Biosystems) [6].

### Western Blot Analysis

Mouse kidney total protein (25μg), and renal brush border membrane vesicles (BBMV)(25μg) were isolated [13,14] and fractionated on 8% SDS-polyacrylamide gel as previously described [11]. For detection of Npt2a and Npt2c proteins, membranes were probed with a rabbit anti-Npt2a polyclonal antibody (1:300) (Alpha Diagnostic International, Inc., San Antonio, TX) and rabbit anti-Npt2c polyclonal antibody (1:1000) (Sigma-Aldrich, St. Louis, MO). Equal protein loading was determined using a rabbit anti-β-actin polyclonal antibody (1:5000) (Cell Signaling Technology, Danvers, MA). The membranes were subsequently blotted using an infrared (IR) labeled secondary antibody (Li-Cor Biosciences, Lincoln, NE). The bound complex was detected using Odyssey Infrared Imaging System (LiCor Biotechnology).

### Chromatin-immunoprecipitation and genome wide sequencing

Chromatin was prepared from mouse kidney as per standard protocol (2 kidneys from each mouse were used to prepare one chromatin sample). 30μg chromatin and 3.4 μg of rabbit monoclonal anti-Egr-1 antibody (Cell Signaling Technology, Danvers, MA) were used for the ChIP experiments. Vehicle-treated mouse kidneys were used as a biological negative control. Prior to sequencing, we validated the egr-1 ChIP samples by ChIP-QPCR using Nab1 primers (positive control for egr-1 binding) and normalized to ChIP samples with control IgG and untreated chromatin (negative controls). Induction of Egr-1 binding to Nab1 was 10-fold higher in FGF23-treated samples compared to vehicle-treated samples (data not shown). DNA libraries were subsequently prepared and amplified after ChIP and submitted for sequencing on the Illumina Hi-Seq platform (Active Motif, CA). Genomic regions with local enrichments in signal intensity (“peaks”) were identified using the MACS algorithm method [15] that looks for significant enrichments in the ChIP data file when compared to the Input data file (i.e. random background). MACS is suitable to identify the binding sites of transcription factors with known binding motifs. Peaks are reported as intervals that have a start and end coordinate. The length of each interval varies from 500 bp to 1500 bp. Intervals present in Input or IgG control samples (i.e. false peaks) were used to identify false positives in egr-1 ChIP samples. To compare peak metrics between 2 samples, we grouped overlapping intervals into “Active Regions”, which are defined by the start and end coordinate of the most upstream and downstream interval, respectively (= union of overlapping intervals). In locations where only one sample has an interval, this interval defines the active region. After defining the active regions, their exact locations along with their proximities to gene annotations are reviewed using the data visualization tools on the UCSC (http://genome.ucsc.edu) and IGB genome browsers (http://bioviz.org/igb/download.html). False discovery rates were calculated and were noted to be low at less than 0.1%. Approximately 9 million sequence tags were uniquely mapped to the genome per sample. After normalization, we obtained ~ 9,000 and 7,000 unique active regions for FGF23-treated, 1 and 2 hr samples, respectively. To refine the *in vivo* consensus egr-1 motif, we queried MEME (a web-based tool to identify transcription factor binding motifs) [16,17] with the top 1,200 enriched regions corresponding to the most statistically significant binding sites identified by ChIP-seq. Additional transcription factor motifs that were identified in the vicinity of egr-1 binding are shown in S1 Table. We validated the results of the ChIP-seq dataset using the Nab1 gene as a positive control. This active region (Nab 1-AR) was further shown to contain an enhancer element that is responsive to FGF23 in HEK293 cells transfected with the Nab 1-AR sequence upstream of the luciferase reporter gene (S1 Fig).

### Microarray and Pathway Analysis

Microarray analysis was performed on kidneys from 6 week-old mice treated with FGF23 or vehicle for 1 hr. Total RNAs were isolated using TRIreagent (Molecular Research Center, Cincinnati, OH, USA). The expression of 45,000 genes was tested on the kidney samples using the Illumina.SingleColor. Mouse WG-6_V2_0_R1_11278593_A chip (Illumina, San Diego, CA, USA) at the DNA Discovery Core of University of Tennessee Health Science Center. Data were analyzed using GeneSpring GX software (Agilent Technologies, Santa Clara, CA, USA). The Robust Multichip Averaging probe summarization algorithm was used to perform background correction, normalization, and probe summarization. Microarray data were normalized per chip and per gene to the median. Genes were filtered to include only those that were expressed in at least one of the samples. The statistical analysis was performed using Mann-Whitney unpaired test with a p value cutoff of p<0.05 followed by Benjamini-Hochberg multiple test correction to minimize the false positive discovery rate (FDR). The resulting data were compared with our ChIP-seq data and previously published data reflecting the renal transcriptome in Col4a3^-/-^ [18] and FGF23 transgenic mice [19] with chronic FGF23 excess. Pathway analysis was performed using the Ingenuity program (Ingenuity Systems, Redwood City, CA, USA) to match the identified genes of interest to already known broader networks of genes contained in the literature database.

### Cell Culture and Transfection

Human embryonic kidney (HEK-293) cells (a kind gift from Makoto Kuro-o, University of Texas Southwestern, Dallas, TX) were stably transfected with the transmembrane (Tm) form of mouse klotho-pEF1 expression vector and maintained as previously described [5]. Klotho is an obligatory co-factor for FGF-23 and confers tissue specificity for it actions in target tissues [4]. It is well established that FGF-23-dependent signal activation in HEK-293 cells is dependent on Tm klotho [4,5]. HEK-293 cells were plated at 130,000/well in 24-well plates, in DMEM H-21 with 10% FBS (Hyclone, Waltham, MA). At 80% confluence, HEK293 cells were transfected with a thymidine kinase (TK) promoter-driven luciferase plasmid containing Nab 1-active region (AR) (930bp in length) and treated with FGF23 (100ng/ml) or vehicle for 24hrs. Luciferase activity was normalized to the empty vector and expressed as relative luciferase units (RLU).

### Statistical Analysis

Data are expressed as means ± SEM. The significance of differences between vehicle and treatment groups was analyzed by ANOVA or t-test when appropriate, using Sigma Stat statistical software (Jandel Scientific, San Rafael, CA).

## Results

### Effects of egr-1 gene deletion on Pi homeostasis in mice

**Fig 1:**
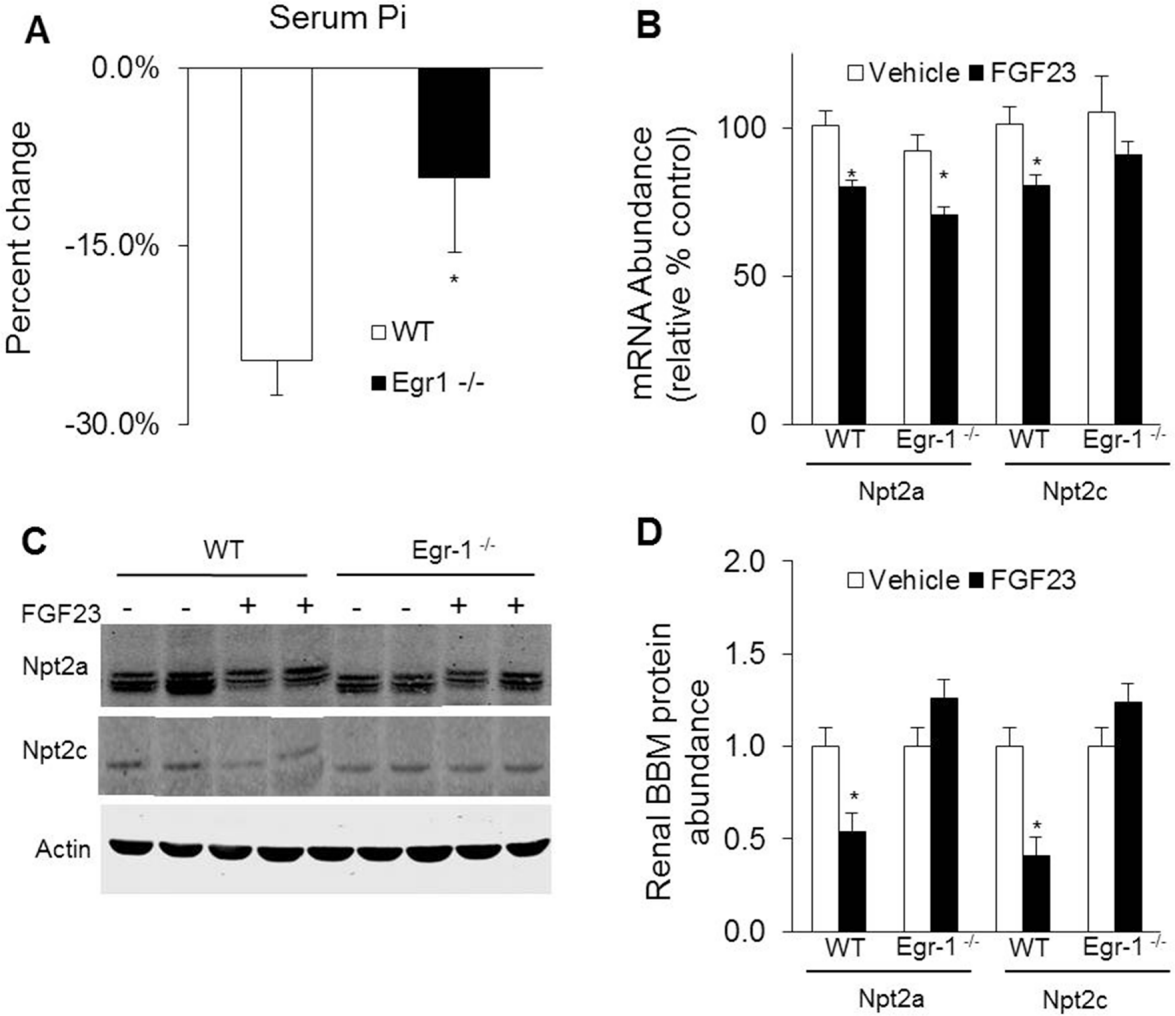
Effects of egr-1 gene deletion on phosphate homeostasis. *Egr-1* ^*-/-*^ and wild-type (WT) mice were treated with vehicle or FGF23. *A*. Serum phosphorus concentration. Bars depict percent change when compared to vehicle treated group (n = 8–10 mice/group). *B*. Renal Npt2a and Npt2c mRNA abundance were quantitated by real-time PCR, normalized to that of gus mRNA, and expressed as a percent relative to vehicle-treated mice. Bars depict mean ± SEM (n = 8–10 mice/group) * *P<0*.*05*, compared to *egr-1* ^*-/-*^ and WT mice treated with vehicle. *C* and *D*. Renal Npt2a and Npt2c protein abundance in renal brush border membrane vesicle preparations normalized to β-actin.

To determine whether egr-1 mediates the phosphaturic effect of FGF23, we administered FGF23 intraperitoneally to *egr-1* ^*-/-*^ and WT mice. At baseline, the mean serum Pi concentrations were not significantly different in *egr-1* ^*-/-*^ mice when compared to WT mice. After treatment with FGF23, serum Pi concentration decreased by 27% in WT mice (8.3 ± 0.4 vs 6.1 ± 0.3 mg/dl, *P<0*.*05*) and by 12% in *egr-1* ^*-/-*^ mice (7.4 ± 0.5 vs 6.5 ± 0.3 mg/dl, *P = 0*.*06)*. Thus, the hypophosphatemic response to FGF23 was blunted by 56% in *egr-1* ^*-/-*^ mice (Fig 1A). We then determined the renal mRNA and protein abundance of the NaPi cotransporters, Npt2a and Npt2c. At baseline, renal abundance of Npt2a and Npt2c mRNA and protein were not significantly different between the two groups. After treatment with FGF23, Npt2a mRNA expression decreased significantly in both groups of mice by approximately 22% (*P<0*.*05*). However, Npt2a protein abundance in renal brush border membrane (BBM) preparations decreased only in WT mice and was unchanged in *egr-1* ^*-/-*^ mice. The renal abundance of both Npt2c mRNA and protein decreased significantly only in FGF23-treated WT mice but were unchanged in FGF23-treated *egr-1* ^*-/-*^ mice (Fig 1B–1D).

### Effects of egr-1 gene deletion on vitamin D homeostasis in mice

**Fig 2:**
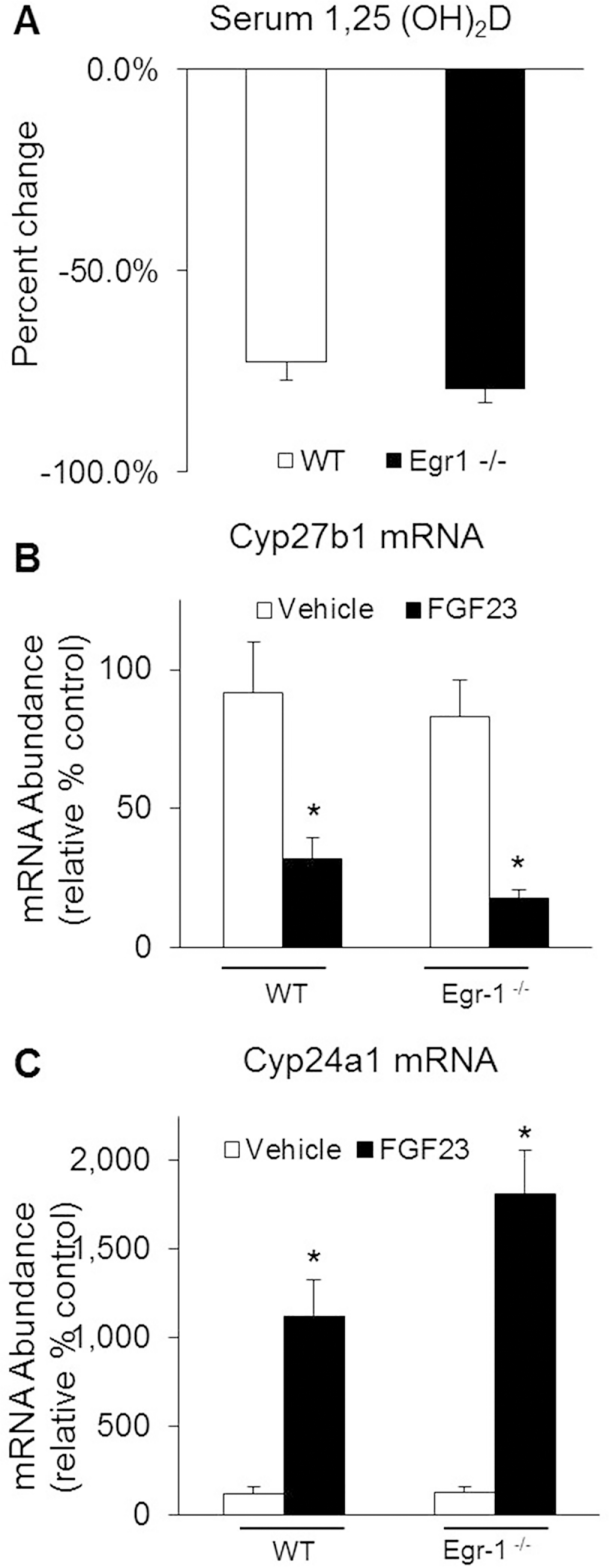
Effects of egr-1 gene deletion on renal 1,25(OH)_2_D metabolism. *Egr-1* ^*-/-*^ and wild-type (WT) mice were treated with vehicle or FGF23. *A*. Serum 1,25(OH)_2_D concentrations. *B*. Renal c*yp27b1* and, *C*. Renal *cyp24a1* mRNA abundance were quantitated by real-time PCR, normalized to that of gus mRNA, and expressed as a percent relative to vehicle-treated WT mice. Bars depict mean ± SEM (n = 8–10 mice/group) * *P<0*.*05*, compared to *egr-1* ^*-/-*^ and WT mice treated with vehicle.

**Fig 3:**
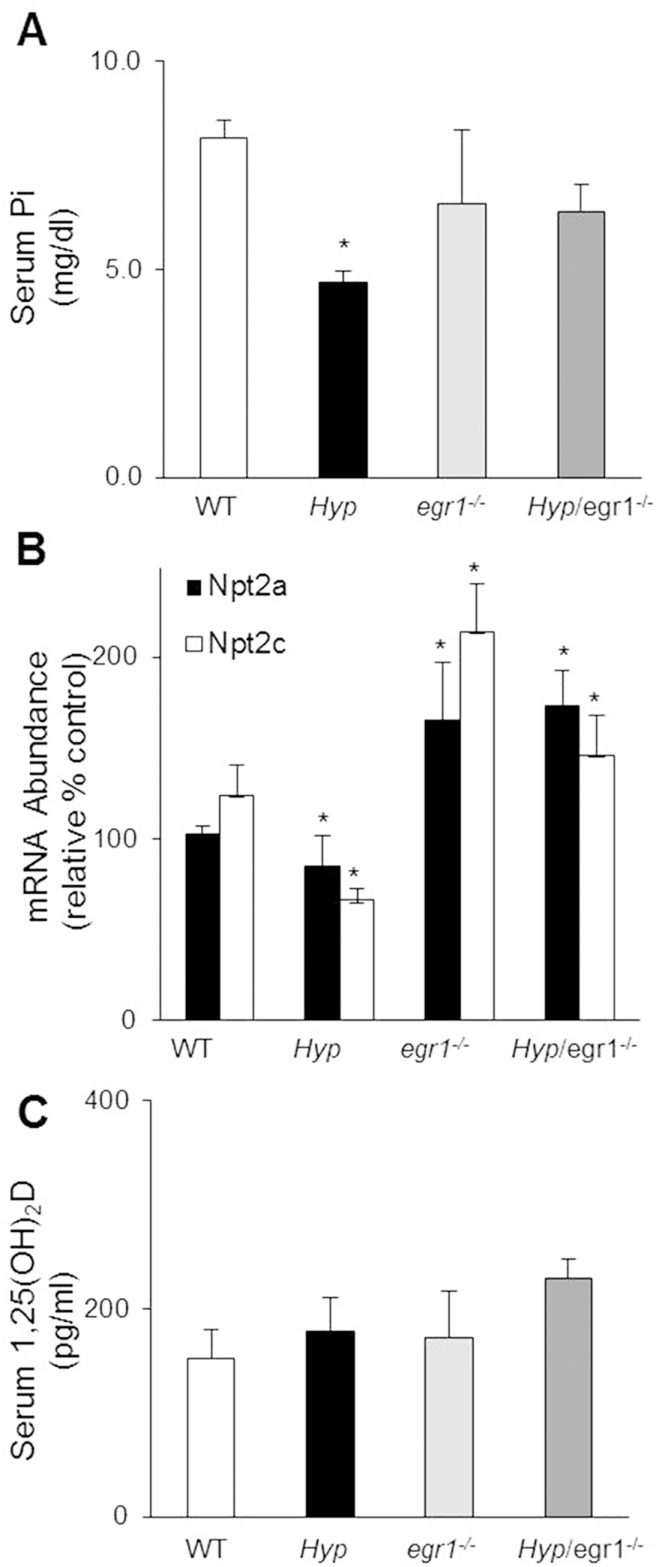
Effects of egr-1 gene deletion on phosphate homeostasis in *Hyp* mice. WT, *Hyp*, *egr-1* ^*-/-*^ and *Hyp/egr-1* ^*-/-*^ mice were sacrificed at 7–10 weeks of age to determine the following: *A*. Serum phosphorus concentration. *B*. Renal Npt2a and Npt2c mRNA abundance were quantitated by real-time PCR, normalized to that of gus mRNA, and expressed as a percent relative to WT mice. *C*. Serum 1,25 (OH)_2_D concentration. Bars depict mean ± SEM (n = 5 mice/group) * *P<0*.*05*, compared to WT mice.

To determine the effect of egr-1 gene deletion on vitamin D homeostasis, we measured serum 1,25(OH)_2_D concentrations and the renal abundance of c*yp27b1* and *cyp24a1* mRNA (Fig 2A–2C). At baseline, the mean serum 1,25(OH)_2_D concentration in *egr-1* ^*-/-*^ mice was not significantly different from that in WT mice (Fig 3A). FGF23 treatment induced significant suppression of serum 1,25(OH)_2_D concentrations in both groups of mice (Fig 2A). At baseline, abundance of *cyp27b1* and *cyp24a1* mRNA in *egr-1* ^*-/-*^ mice was not significantly different from those in WT mice. With FGF23 treatment, c*yp27b1* expression was greatly suppressed and *cyp24a1* mRNA was greatly increased in both groups of mice (Fig 2B and 2C). The mean serum calcium and PTH concentrations did not differ between *egr-1* ^*-/-*^ and WT mice in the vehicle-treated group or after FGF23 treatment (data not shown). Thus, FGF23 can regulate vitamin D metabolism normally even in the absence of egr-1 expression.

### Effects of egr-1 gene deletion on Pi homeostasis in Hyp mice

*Hyp* mice exhibit phosphaturia and hypophosphatemia due to excess circulating FGF23 and this hypophosphatemic effect is completely rescued when the fgf23 gene is deleted in *Hyp/fgf23* ^*-/-*^ mice [20,21]. To determine whether egr-1 mediates the hypophosphatemia induced by FGF23 in *Hyp* mice, we generated the double mutant *Hyp*/*egr-1* ^*-/-*^ mice. In *Hyp*/*egr-1* ^*-/-*^ mice, serum Pi concentrations were not significantly different from *egr-1* ^*-/-*^ or WT mice (Fig 3A). As expected, serum Pi concentrations were 42% lower in *Hyp* mice than in WT mice, consistent with previous reports. Renal mRNA abundance of Npt2a and Npt2c were lower in *Hyp* mice when compared to WT mice and this effect was completely reversed by ablation of the egr-1 gene in *Hyp*/*egr-1* ^*-/-*^ mice (Fig 3B). The mean serum 1,25(OH)_2_D, calcium and PTH concentrations in *Hyp*/*egr-1* ^*-/-*^ mice were not significantly different from those in *egr-1* ^*-/-*^ or WT mice (Fig 3C and S2 Table).

### Characterization of the egr-1 cistrome in mice kidney

**Fig 4:**
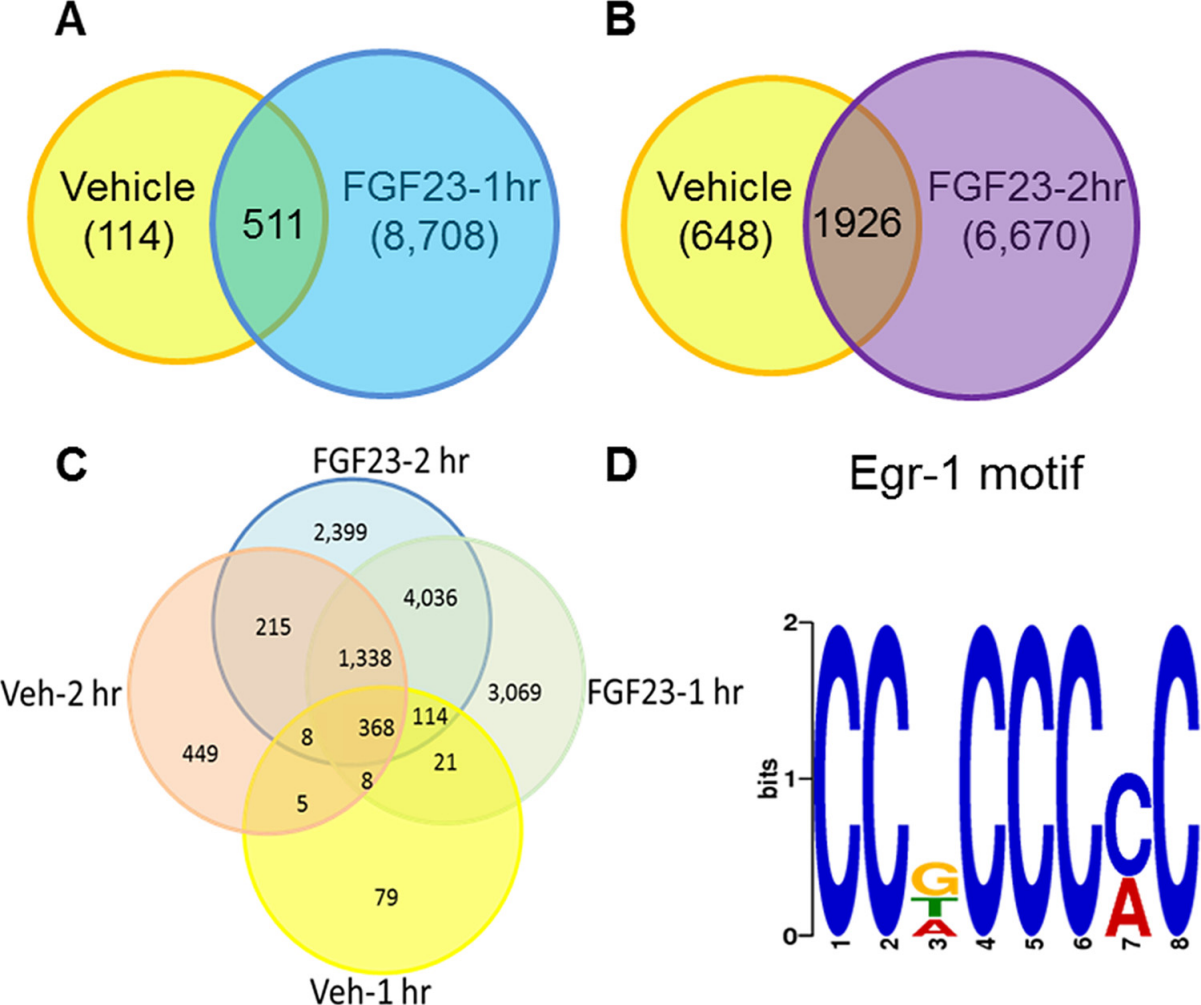
Schematic representation of the active regions identified by ChIP-seq analysis in normal mice kidney after treatment with FGF23 or vehicle. *A-C*. Venn and Euler diagrams showing overlap of egr-1 binding sites between different treatment groups. *D*. Motif discovery using MEME. Consensus motif for egr-1, as determined by querying the 1,200 most enriched ChIP-seq loci.

To further explore the molecular mechanisms responsible for the blunted hypophosphatemic response to FGF23 treatment in mice lacking the egr-1 gene, we sought to obtain a comprehensive view of the genes regulated by FGF23 downstream of egr-1. To define the egr-1 cistrome (i.e genome-wide location of transcription factor (egr-1) binding-sites) in the kidney, we performed ChIP-seq analysis in WT mice treated with FGF23 or vehicle. We obtained unique DNA sequences or “active regions” across the mouse genome (Fig 4A–4C). We constructed an Euler diagram to display the active regions identified in the 4 groups of mice. 43% of the unique active regions were identified in both treatment groups, 32% and 25% of the active regions were unique to the 1hr and 2hr treatment groups, respectively (Fig 4C). The top consensus motif in our dataset identified using MEME software tool was indeed the egr-1 consensus sequence 5'-CCG CCC CC-3' (Fig 4D), thereby validating the ChIP-seq experiment. This motif is identical to the most overrepresented sequence identified in a recent egr-1 ChIP-seq study of mouse brain [22], and is a close match to the canonical egr-1 DNA response element previously characterized [23].

**Fig 5:**
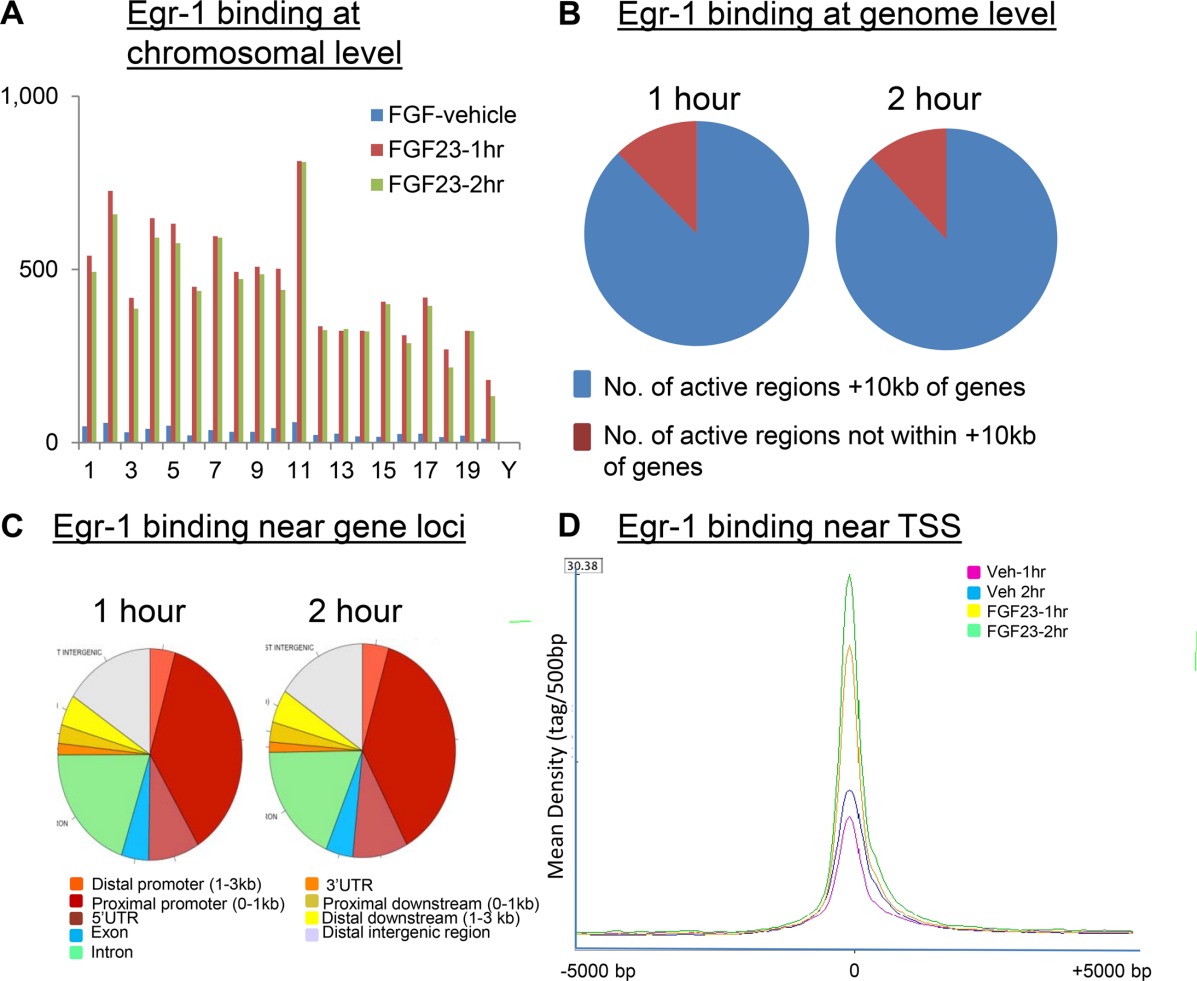
Analysis of the egr-1 cistrome. Genome-wide distribution of egr-1 binding sites in normal mice kidney after treatment with FGF23 or vehicle. *A*. Visualization of egr-1-binding sites across all mouse chromosomes. *B*. Spatial distribution of egr-1 binding sites at the genome level and, *C*. near individual genes. *D*. The ChIP-seq and input data files were analyzed by seqMINER, which analyzes the signal profiles across mouse promoters (TSS), and clusters the profiles according to binding patterns. The result shows that the 3 clusters with the strongest signals (vehicle, FGF23 -1hr and FGF23-2hr) contained 1328, 3288, and 4811 genes (total = 9427), respectively. Vehicle and FGF23-treated samples did not show a difference in signal profile but differed markedly in signal intensity.

We next examined the distribution of egr-1 binding sites across the mouse genome in FGF23-treated mice. The locations and amplitudes of peaks were strikingly similar between 1 hr and 2 hr samples in FGF23-treated mice; similarity in egr-1 binding was seen at the chromosomal level (Fig 5A) and genome wide level (Fig 5B and 5C). The majority (88%) of egr-1-bound regions mapped within 5 kb of transcription start sites (TSS) (Fig 5D), which is consistent with reports showing that transcription factors prefer to bind in the vicinity of genes [24]. The distribution of the egr-1 binding sites near individual gene loci was further analyzed and is depicted in Fig 5C. We discovered a striking enrichment for egr-1-binding sites (41 and 42% for the FGF23-treated 1 hr and 2 hr samples, respectively) in the proximal promoter (0–1kb) region of individual gene loci. A significant number of binding sites were also observed in the introns and distant intergenic region (Fig 5C).

### Characterization of egr-1 binding to active regions near genes responsible for renal Pi transport

To further analyze the role of egr-1 in FGF23-mediated inhibition of renal Pi transport, we queried the egr-1 ChIP seq database for genes encoding proteins reported to participate in intracellular Pi transport in the kidney (i.e. cotransporters, scaffold and trafficking proteins). In mice treated with FGF23, we found a 2- to 16- fold increase in egr-1 binding near genes encoding for Na/Pi cotransporters (Npt2a and Npt2c), scaffold proteins (NHERF1-3, ezrin and GABARAP), and trafficking proteins (megalin and vacuolar ATPase). Several of the active regions we identified were found in the core promoter region which strongly suggests that these genes are transcriptionally regulated by FGF23 (Table 1). Although egr-1 gene ablation did not alter the suppressive effect of FGF23 on Npt2a mRNA in egr-1^-/-^ mice, we identified four active regions bound by egr-1 near the Npt2a gene. Of note, PDZK2, Pex-19, Shank2E and cortactin were not bound by egr-1.

**Table 1:** List of genes present in the CHIP-seq datasets and reported in the literature to be involved in renal Pi transport.

Gene Name	Known Function	Location of AR (Distance from TSS)	Length of AR (bp)	Average Fold Change*
Slc34a1 (Npt2a)	Co transporter	-7746,159, 9,260,15434	777, 691, 1092,752	2
Slc34a3 (Npt2c)	Co transporter	5576, 9505,10401	2268, 881, 521	3
Slc9a3r1 (NHERF-1)	Scaffolding protein	53,1641, 15829, 18715,24077	910,1577, 884,848, 481	3
Slc9a3r1 (NHERF-2)	Scaffolding protein	259,17633	1223, 499	16
PDZK1 (NHERF-3)	Scaffolding protein	-24	482	2
MAP17 (PDZK1P1)	Scaffolding protein	2209	617	2
Ezrin (Vil2)	Scaffolding protein	-295, 40534, 51001, 54448	808, 791, 1377,713	8
GABARAP	Scaffolding protein	-66,5101,9248	1172, 2573, 729	3
Megalin (LRP2)	Protein trafficking	-65	735	6
Vacuolar ATPase (ATP6V1A)	Protein trafficking	-426	1620	2
Pex-19	Scaffolding protein	-	-	Not detected
PDZK2 (NHERF-4)	Scaffolding protein	-	-	Not detected
Shank2E	Scaffolding protein	-	-	Not detected
Cortactin	Scaffolding protein	-	-	Not detected

*Fold change represents egr-1 binding events in FGF23 treated 1-hr sample when compared to vehicle-treated sample. AR-Active Region, TSS-Transcription start site

### Identification of direct Egr-1 target genes

**Fig 6:**
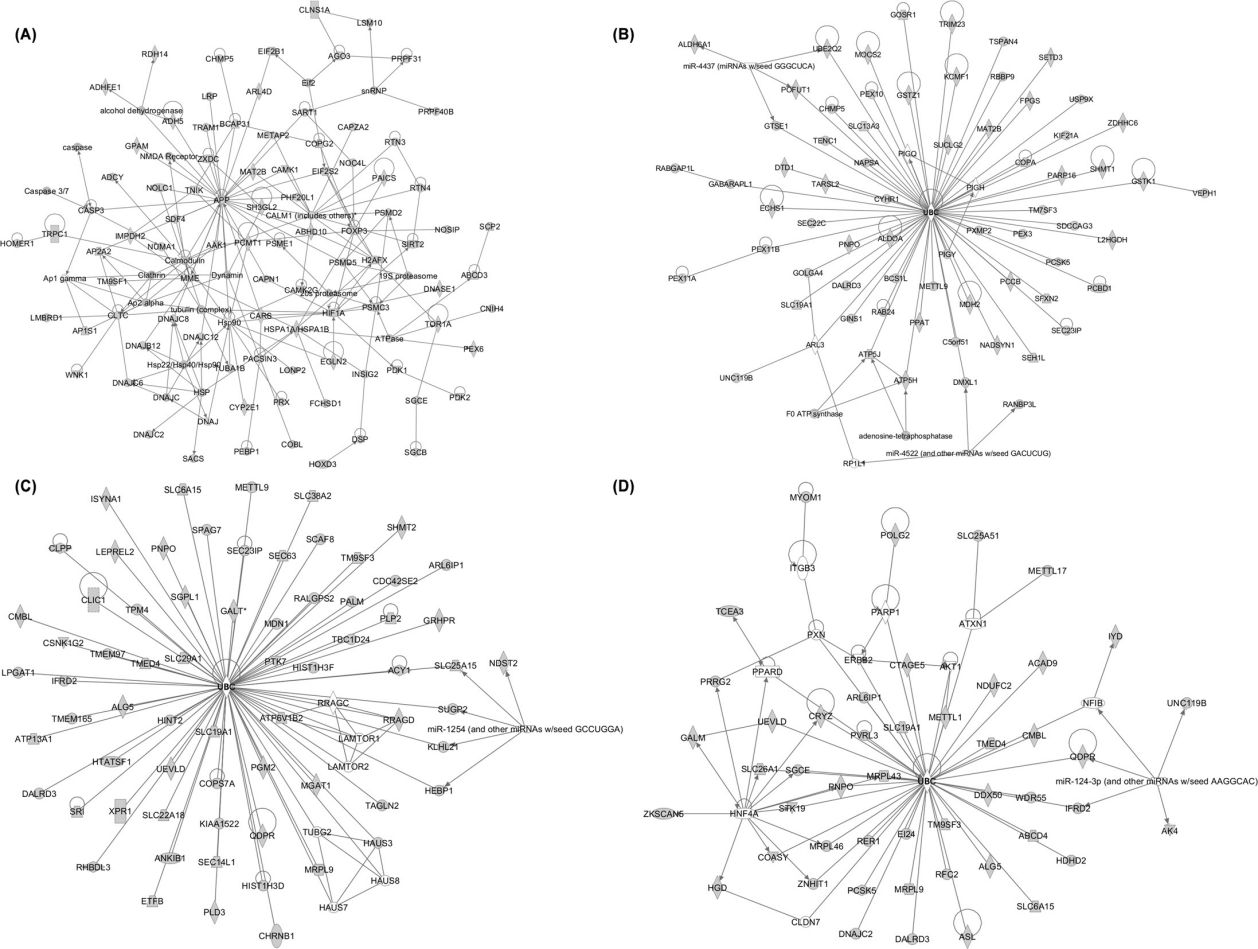
Ingenuity Pathway analysis (IPA)-Top regulatory networks representing genes involved in cellular assembly and organization. *A*. Network of common genes found in CHIP-seq and microarray datasets. Ubiquitin C (UBC) shown as the central regulator. *B*. Network of common genes found in CHIP-seq, microarray and Col4a3^-/-^ mice datasets, *C*. Network of common genes found in CHIP-seq and FGF23^Tg^ datasets and, *D*. Network of common genes found in microarray and FGF23^Tg^ datasets. The networks are built according to the identified interconnected pathways involving the majority of genes displaying direct interactions. Genes represented in gray belong to the dataset. Genes represented in white are intermediary regulators that do not belong to the cluster.

We next determined “direct” egr-1 target genes; i.e., genes that are both bound by egr-1 and actively transcribed in the kidney after FGF23 treatment. To this end, we performed RNA microarray analyses on kidney tissue from mice treated with FGF23 for 1hr and intersected the microarray and the ChIP-seq data for the same time point. The list of genes with the highest fold change in the microarray analysis is provided in S3 Table. Based on the expression patterns from the microarray analysis and using a cutoff value of p<0.05, we found 1,425 genes common to both datasets; of these, 120 (8%) were up-regulated and 1,305 (92%) were down-regulated by egr-1 after FGF23 injection. The list of genes with the highest fold change in the intersection analysis is shown in S4 Table. We then performed an Ingenuity Pathway Analysis (IPA) on 1,425 genes. IPA categorized the genes into 5 top molecular and cell functions and 3 top regulatory networks of equal importance (S5 Table). “Cellular assembly and organization” was identified in both networks and function categories, and connections between genes from this network were mapped in Fig 6A. In this category, several members of the calmodulin family were among the most interconnected genes in the network and therefore represented valid candidates for FGF23-regulated gene targets via egr-1. Consistent with this finding, previous studies have shown that the calmodulin-dependent protein kinase pathway is involved in Pi transport [25,26]. Our findings suggest that FGF23 activates the calmodulin pathway to inhibit renal Pi transport.

To further elaborate the genes regulated by FGF23/egr-1 signaling axis in conditions of both acute and chronic elevations of serum FGF23, we did the following bioinformatics analyses; we intersected the set of genes common to Chip-Seq and microarray datasets with available microarray expression profiles reported previously from kidney samples of mice with FGF23 excess (Alport’s chronic kidney mouse model (Col4a3^-/-^), and FGF23 transgenic (FGF23^Tg^) mice) [18,19]. Intersection with the Col4a3^-/-^ dataset identified 222 genes in common with the Chip-Seq & Microarray dataset. Down-stream IPA analyses of the 222 genes, revealed a single top regulatory network out of 4 significant networks computed. This network was formed by genes involved in “cellular assembly and organization” (S6 Table), with ubiquitin C as a central network regulator. This rare occurrence, of a single central network organizing gene, reflects that protein ubiquitinylation by ubiquitin C is the only known common mediator between all the regulated genes (Fig 6B). Individual intersection analysis identified 1,406 and 501 genes in common between Col4a3^-/-^ and Chip-Seq, and Col4a3^-/-^ and microarray datasets, respectively. Separate IPAs were performed on each group of genes and “cellular assembly and organization” was identified again as the top regulatory network. A similar approach was used to intersect the common genes from the Chip-seq and microarray datasets with the FGF23^Tg^ database. Intersection of the FGF23^Tg^ dataset identified 468 and 131genes in common with the Chip-Seq and microarray datasets, respectively. Ubiquitin C was consistently found as a single factor connecting the gene network (Fig 6C and 6D). Thus, cellular assembly and organization network, and ubiquitin C were consistently identified in the intersection of four genomic datasets associated with FGF23 excess.

### FGF23/Egr-1 signaling down-regulates vesicle trafficking proteins in the kidney

In the mouse brain, a significant number of egr-1 target genes are strongly associated with vesicle transport [22]. Since vesicle trafficking is critical for movement of ion transporters to and from the proximal tubule brush border membrane, we next sought to determine whether vesicle trafficking proteins in the kidney are regulated by FGF23/egr-1 signaling. We queried the human vesicle trafficking protein database [27] (a mouse database is currently not available). Using a 2-fold cut off for increased egr-1 binding, we identified 102 genes from our ChIP-seq dataset that overlap with the vesicle trafficking database. We also performed a Gene Set Enrichment Analysis (GSEA) with a cutoff of p<0.05 and a false discovery rate (FDR) q-value of p<0.1, and found that 47 vesicle trafficking genes were bound by egr-1 following FGF23 treatment (data not shown). We then intersected the egr-1 ChIP-seq data with the microarray data for the same time point (1hr) to obtain a list of vesicle trafficking genes that are both bound by egr-1 and whose gene expression is regulated by FGF23. Using GSEA with the same established parameters, we found that 14 vesicle genes were down-regulated in both databases (Table 2) including two lectins (ERGIC-1 and -3), SNX9, a membrane remodeling protein which interacts with actin-regulating proteins [28], the lysosomal trafficking protein VPS41 and synaptophysin.

**Table 2:** List of vesicle trafficking genes that are present in both CHIP-seq and microarray datasets.

Gene Name	Symbol	Fold Change*
DownRegulated Genes		
B-cell receptor-associated protein 31	BCAP31	-1.7
sorting nexin 9	SNX9	-1.7
golgi SNAP receptor complex member 1	GOSR1	-1.6
transmembrane emp24 protein transport domain containing 4	TMED4	-1.6
endoplasmic reticulum-golgi intermediate compartment 1	ERGIC1	-1.5
SH3-domain GRB2-like 2	SH3GL2	-1.5
syntaxin 18	STX18	-1.4
vacuolar protein sorting 41	VPS41	-1.4
adaptor protein complex AP-2, alpha 2 subunit	AP2A2	-1.3
adaptor-related protein complex AP-4, epsilon 1	AP4E1	-1.3
endoplasmic reticulum-golgi intermediate compartment 3	ERGIC3	-1.3
synaptophysin	SYP	-1.3
S. cerevisiae vesicle trafficking protein-like C 22	SEC22C	-1.2
AP2 associated kinase 1	AAK1	-1.1

*Fold change represents change in gene expression in microarray datasets in FGF23 treated 1-hr sample when compared to vehicle-treated sample. Fold change in ChIP-seq datasets not shown.

### DNase I: A novel mechanism for FGF23 to regulate renal Pi reabsorption

**Fig 7:**
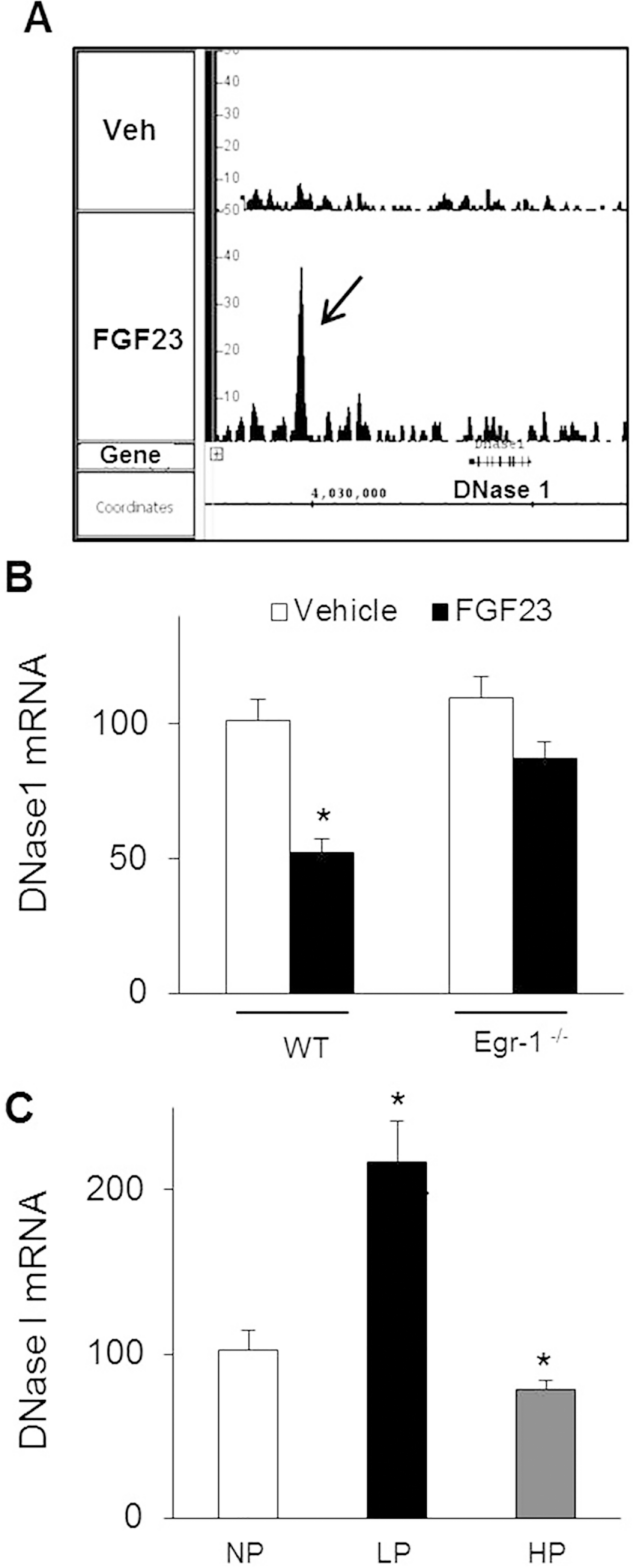
Renal DNase I gene expression. A. Egr-1 ChIP-seq analysis showed recruitment of egr-1 to DNase I. Peak signal intensity for egr-1 binding up-stream (-7700 bp) of DNase I gene in FGF23-treated mice. A low signal is noted in the vehicle-treated sample. *B*. Renal DNase I mRNA abundance in WT and *egr-1* ^*-/-*^ mice treated with vehicle or FGF23. *C*. Renal DNase I mRNA abundance in normal mice fed a normal (NP), low (LP) or high (HP) Pi diet for 5 days. Bars depict mean ± SEM (n = 5 mice/group). * P < 0.*05* compared to control group.

By intersecting all 4 databases that reflect FGF23 excess (Chip-seq, microarray, Col4a3^-/-^ and FGF23^Tg^) using IPA, we identified 18 down-regulated genes (Table 3), among which DNase I was one of the top candidates. Interestingly, DNase I was previously found to be one of the top down-regulated genes in the kidney of *Hyp*, FGF23^Tg^ and Col4a3^-/-^ mice [18,19,29]. Here, we demonstrate that egr-1 binds to an active region ~ -7700 bp from the transcription start site of DNase I gene (Fig 7A). To study the functional role of DNase I, we determined whether DNase I gene expression is regulated by FGF23 and by dietary Pi intake. Normal mice were either injected with FGF23 or fed a high (1.65%), or low (0.02%) Pi diet for 5 days. DNase I mRNA expression was significantly suppressed by FGF23 injection (47%) and by high Pi diet (20%), whereas a low Pi diet induced a 200% increase in DNase I mRNA expression (Fig 7B and 7C). Furthermore, FGF23 failed to suppress renal DNase I mRNA expression in egr-1^-/-^ mice (Fig 7B). These studies demonstrate that DNase I is indeed regulated by FGF23 and dietary Pi intake, and may play a physiologic role in Pi homeostasis.

**Table 3:** List of genes down regulated in Chip-seq, microarray, Col4a3^-/-^ and FGF23^Tg^ datasets.

Gene Name	Symbol	*Fold Change
DownRegulated Genes		
Adenylate kinase isoenzyme 4	AK3L1	-2.0
Deoxyribonuclease I	DNASE1	-1.6
Estrogen-related receptor alpha	ESRRA	-1.6
Zinc finger with KRAB and SCAN domains 5	ZKSCAN5	-1.6
Prostasin	PRSS8	-1.5
Proprotein convertase subtilisin/kexin type 5	PCSK5	-1.5
Autocrine motility factor receptor, E3 ubiquitin protein ligase	AMFR	-1.5
Nucleosome assembly protein 1-like 1	NAP1L1	-1.5
Peroxisomal biogenesis factor 11 alpha	PEX11A	-1.5
Solute carrier family 19 (folate transporter), member 1	SLC19A1	-1.5
Homogentisate 1,2 dioxygenase	HGD	-1.4
Glutathione S-transferase zeta 1	GSTZ1	-1.4
Lon peptidase 1	LONP1	-1.4
DALR anticodon binding domain containing 3	DALRD3	-1.4
Unc-119 Homolog B	UNC119B	-1.2
Pyridoxamine 5'-phosphate oxidase	PNPO	-1.1
Androgen receptor	AR	-1.1
5-azacytidine-induced protein 2	AZI2	-1.1

*Fold change represents change in gene expression in microarray datasets in FGF23 treated 1-hr sample when compared to vehicle-treated sample.

## Discussion

In the present study, we provide evidence that egr-1 mediates FGF23-dependent inhibition of renal Pi transport but plays no apparent role in FGF23-mediated inhibition of 1,25(OH)_2_D synthesis. Further, we characterize the FGF23-dependent egr-1 cistrome in the kidney. To our knowledge ours is the first study to identify a transcription factor, egr-1, that mediates the hypophosphatemic effect of FGF23 and to differentiate the effector pathways that regulates inhibition of renal Pi reabsorption from that of 1,25(OH)_2_D synthesis. Using genome-wide ChIP-sequencing, we have identified components in the Pi transport network that are regulated by the FGF23/egr-1 signaling axis, including putative novel targets with protein trafficking function.

Previously, we showed that systemic inhibition of MEK/ERK1/2 signaling in *Hyp* mice improved hypophosphatemia by 70% [10,11]. In the present study, we demonstrate that deletion of the egr-1 gene, which is downstream of MEK/ERK1/2 signaling, blunts the hypophosphatemic response to acute administration of FGF23 by approximately 56% in *egr-1* ^*-/-*^ mice. In *Hyp*/*egr-1* ^*-/-*^ mice which have chronically high circulating FGF23 concentrations, deletion of the egr-1 gene improves hypophosphatemia by 50% when compared to *Hyp* mice. We also observed that suppression of Na/Pi cotransporter abundance was blocked in *egr-1* ^*-/-*^ mice treated with FGF23 and in *Hyp*/*egr-1* ^*-/-*^ mice. Furthermore, using bioinformatics we demonstrate that egr-1 transcriptionally regulates the gene expression of scaffold and trafficking proteins that mediate the insertion and retrieval of Npt2a from the BBM as discussed further below.

It is a currently held view that Pi reabsorption is regulated by changing the number of Na/Pi cotransporters that reside in the BBM rather than by changing their intrinsic transport activity [30]. BBM abundance of Npt2a and Npt2c is dependent on their gene transcription, and their insertion and retrieval from the BBM, which in turn is dependent on their interaction with scaffolding and trafficking proteins [31–34]. However, little is known about FGF23-induced inhibition of renal Pi reabsorption at the cellular level. We and others have shown that FGF23 inhibits BBM Pi uptake via suppression of Npt2a and Npt2c mRNA and protein abundance [6,8]. The specific role for NHERF-1 in FGF23-induced inhibition of Pi transport was also recently demonstrated [35,36]. To investigate the interaction of the cotransporters with intracellular scaffolding and trafficking proteins, we utilized a novel bioinformatics approach to expand our limited knowledge of the Pi transport network in the context of FGF23 action. We identified several novel putative gene targets for FGF23, including those that encode a subset of scaffold and vesicle trafficking proteins in the kidney (Tables 1 and 2). Several of the active regions identified were within the proximal promoter region (0–1kb) including binding sites for ezrin, GABARAP, megalin, vacuolar ATPase and NHERF-3. Interestingly, egr-1 bound to genes encoding scaffolding proteins, NHERF1, 2 and 3 of which NHERF-2 showed the strongest binding at 16-fold when compared to controls. Of note, several of the genes encoding vesicle trafficking proteins were significantly down regulated by FGF23. Given the rapid transcriptional regulation of these vesicle trafficking genes by FGF23 in an egr-1-dependent manner, it is possible that the direct repression of the vesicle genes may be responsible for the reduced insertion and/or endocytic removal of Npt2a and Npt2c from the apical BBM and the consequent phosphaturic effect of FGF23. Further study is needed to precisely map the intracellular Pi transport network that is regulated by FGF23/egr-1 signaling. Interestingly, a recent ChIP-seq study described the role of egr-1 in mouse brain tissue. A significant number of egr-1 target genes in the brain were strongly associated with protein targeting and localization, endocytosis, and vesicle transport [22]. Thus, the similarities in the egr-1 ChIP-seq datasets in mouse kidney and brain suggest that the cellular functions of egr-1 are highly conserved.

We have demonstrated that blockade of MEK/ERK1/2 signaling corrects the aberrant renal 1,25(OH)_2_D synthesis in *Hyp* mice [10,11]. In the present study however, suppression of renal 1,25(OH)_2_D synthesis was intact in egr-1^-/-^ mice treated with FGF23. These findings provide evidence that downstream of FGF receptor binding and ERK1/2 signaling, the pathways diverge such that egr-1 is required for FGF23-dependent inhibition of Pi transport but not for inhibition of 1,25(OH)_2_D synthesis. Identification of the different signaling pathways for these two actions of FGF23 may have important clinical significance in patients with chronic kidney disease (CKD). In pre-dialysis CKD, excess circulating FGF23 induces phosphaturia, an action thought to be helpful in maintaining Pi homeostasis as the glomerular filtration rate declines [37–40]. However, excess FGF23 induces suppression of renal 1,25(OH)_2_D synthesis and reduction in circulating 1,25(OH)_2_D [39,41], actions thought to be critical to the development of secondary hyperparathyroidism [37,38,42,43]. Thus, distinguishing the effector pathways by which FGF23 regulates Pi reabsorption from those of 1,25(OH)_2_D synthesis maybe important to formulate future therapy for patients with CKD.

Our study has the following limitations. We utilized whole kidney samples and thus, interpretation of the data can be confounded by potential cell type-specific differences in egr-1-binding patterns. To overcome this limitation, we analyzed the expression of known genes within the Na/Pi cotransport network whose expression is largely restricted to the proximal tubule. Secondly, we focused our investigations on the kidney but whether egr-1 regulates intestinal Pi transport is unknown. However, FGF23 does not directly regulate intestinal Pi transport; indirect effects are observed due to its suppressive effect on vitamin D metabolism [44–46]. Thus, it is unlikely that egr-1 plays a direct role in the regulation of intestinal Pi transport.

Prior to the present study, few direct gene targets of FGF23 signaling were known. By integrating ChIP-seq and gene expression data, we identified a set of genes regulated by FGF23/egr-1 signaling. Through intersections of multiple genomic datasets we have identified novel genes and regulatory networks for FGF23; specifically, ubiquitin C and DNase I. The occurrence of a single central network regulator (i.e ubiquitin C) emerging from the intersection of 4 large genomic data sets is very rare, and suggests that ubiquitinylation by ubiquitin C is the common mediator between those genes regulated by FGF23. Previous studies have reported that ubiquitinylation of Npt2a in the kidney and Npt2b in the intestine determine their protein abundance in the apical BBM [47,48]. Further studies are needed to elucidate the specific role of ubiquitin C in renal Pi transport. We identified DNase I as another novel candidate gene for regulation of renal Pi transport. In conditions where serum FGF23 concentrations are increased, DNase I gene expression is significantly down regulated as seen in *Hyp* mice and in WT mice fed a high dietary Pi intake or treated with exogenous FGF23. A low Pi diet elicits the opposite effect; serum FGF23 concentrations are lower [7] and DNase I gene expression is 2-fold higher when compared to a normal Pi diet. These findings demonstrate that DNase I is regulated by both FGF23 and dietary Pi intake in normal mice. DNase I bind to the monomeric (G) form of actin and prevent filamentous (F) actin formation. Actin cytoskeleton is known to participate in the endocytic retrieval of Na/Pi cotransporters [49,50] and it is possible that DNase I modulates the stability of the actin cytoskeleton. Further studies are needed to elucidate the specific role of DNase I in renal Pi transport.

In summary, our data demonstrate that the effect of FGF23 on Pi homeostasis in mice is mediated, at least in part, by activation of transcription factor, egr-1. Thus we have distinguished the signaling pathway that mediates FGF23-dependent inhibition of Pi reabsorption from that which mediates inhibition of 1,25(OH)_2_D synthesis. Using genome wide ChIP-sequencing, we have characterized the egr-1 cistrome in the mouse kidney and identified several gene targets downstream of FGF23/egr-1 signaling. This study provides a large genomic database suitable for further exploration and understanding of the molecular basis for disorders of Pi homeostasis associated with FGF23 excess.

## Supporting Information

S1 Fig Validation of egr-1 ChIP-seq analysis. *A*. Signal histogram of the peaks visualized for the positive control gene, Nab1. Integrated Genome Browser (IGB) shots showing egr-1 binding sites near the *Nab1* gene. Binding of egr-1 to the regulatory region up-stream (-1500 bp) of Nab1gene. A low signal is noted in the vehicle-treated sample. B. Peak signal intensity for Nab1 gene in FGF23-treated (1 and 2hr) mice, represented as fold change over vehicle-treated sample. C. HEK 293 cells transfected with Nab 1 active region (930bp)—Luc plasmid and treated with FGF23 (100ng/ml) or vehicle for 24hrs. Luciferase activity was normalized to the empty vector and expressed as relative luciferase units (RLU). Data expressed as Mean ± SEM, n = 3 replicates, *P<0.05.
(JPG)

S1 Table Top10 transcription factor motifs identified in the ChIP-seq dataset using MEME suite. (DOCX)

S2 Table Serum calcium and PTH concentrations in WT, *Hyp*, *egr-1* ^*-/-*^ and *Hyp/egr-1* ^*-/-*^ mice. (DOCX)

S3 Table List of top 20 genes up- and down-regulated by FGF23 by microarray analysis. (DOCX)

S4 Table List of top 20 genes up- and down-regulated by FGF23 in ChIP-Seq and microarray datasets. (DOCX)

S5 Table Ingenuity Pathway Analysis- Intersection of ChIP-seq and microarray datasets. (DOCX)

S6 Table Ingenuity Pathway Analysis- Intersection of Chipseq, microarray and Col4a3^-/-^ mice datasets. (DOCX)
