# Supplementary figures and images for: Characterization of FGF23-Dependent Egr-1 Cistrome in the Mouse Renal Proximal Tubule
Source: PLoS One. 2015 Nov 20;10(11):e0142924. doi: 10.1371/journal.pone.0142924 (PMC4654537; doi:10.1371/journal.pone.0142924)

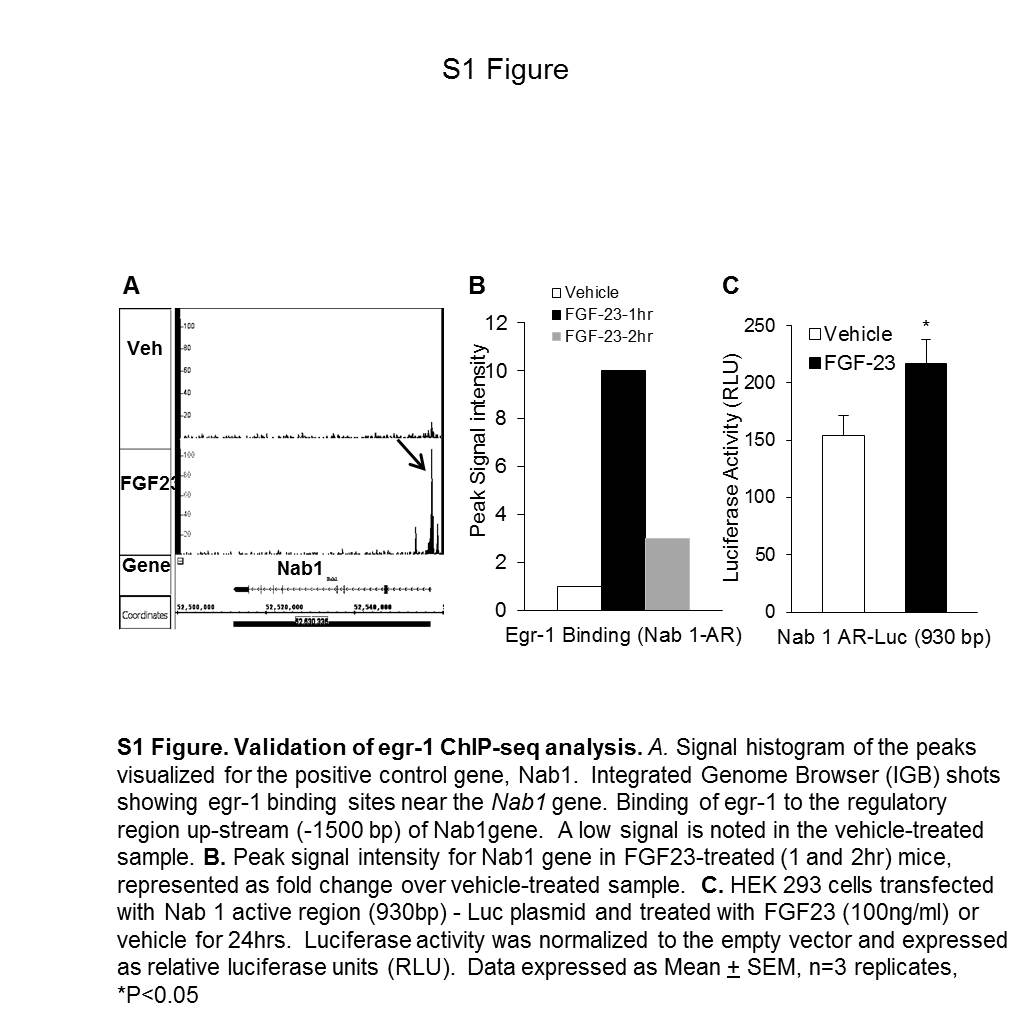

Supplement: S1 Fig — A. Signal histogram of the peaks visualized for the positive control gene, Nab1. Integrated Genome Browser (IGB) shots showing egr-1 binding sites near the Nab1 gene. Binding of egr-1 to the regulatory region up-stream (-1500 bp) of Nab1gene. A low signal is noted in the vehicle-treated sample. B. Peak signal intensity for Nab1 gene in FGF23-treated (1 and 2hr) mice, represented as fold change over vehicle-treated sample. C. HEK 293 cells transfected with Nab 1 active region (930bp)—Luc plasmid and treated with FGF23 (100ng/ml) or vehicle for 24hrs. Luciferase activity was normalized to the empty vector and expressed as relative luciferase units (RLU). Data expressed as Mean ± SEM, n = 3 replicates, *P<0.05. (JPG) [file pone.0142924.s001.jpg]
