# Supplementary material for: Characterization of FGF23-Dependent Egr-1 Cistrome in the Mouse Renal Proximal Tubule
Source: PLoS One. 2015 Nov 20;10(11):e0142924. doi: 10.1371/journal.pone.0142924 (PMC4654537; doi:10.1371/journal.pone.0142924)
Supplement: S1 Table — (DOCX) [file pone.0142924.s002.docx]

**S1 Table.** Top10 transcription factor motifs identified in the ChIP-seq dataset using MEME suite.

| **Transcription Factor** | **P value** |
| --- | --- |
| EGR1 | 2.19647e-10 |
| FLI1 | 2.05771e-05 |
| Mtf1 | 6.95672e-05 |
| EGR2 | 6.04917e-08 |
| STAT1 | 0.000250393 |
| USF1 | 5.24031e-05 |
| NFYB | 1.31896e-05 |
| YY1 | 1.51263e-06 |
| NRF1 | 9.78092e-08 |
| Lmx1a | 1.49936e-05 |
