# Supplementary material for: Characterization of FGF23-Dependent Egr-1 Cistrome in the Mouse Renal Proximal Tubule
Source: PLoS One. 2015 Nov 20;10(11):e0142924. doi: 10.1371/journal.pone.0142924 (PMC4654537; doi:10.1371/journal.pone.0142924)
Supplement: S2 Table — (DOCX) [file pone.0142924.s003.docx]

**S2 Table.** Serum calcium and PTH concentrations in WT, *Hyp, egr-1^-/-^* and *Hyp/egr-1^-/-^* mice.

| **Parameters** | **WT** | ***egr-1^-/-^*** | ***Hyp*** | ***Hyp/egr-1^-/-^*** |
| --- | --- | --- | --- | --- |
| Serum Calcium (mg/dl) | 9.1+0.2 | 8.9+0.2 | 8.4+0.1* | 8.6+0.2 |
| Serum PTH  (pg/ml) | 144+47 | 125+28 | 242+29* | 124+20 |

**P<0.05* when compared to WT mice.
