# Supplementary material for: Characterization of FGF23-Dependent Egr-1 Cistrome in the Mouse Renal Proximal Tubule
Source: PLoS One. 2015 Nov 20;10(11):e0142924. doi: 10.1371/journal.pone.0142924 (PMC4654537; doi:10.1371/journal.pone.0142924)
Supplement: S3 Table — (DOCX) [file pone.0142924.s004.docx]

**S3 Table.** List of top 20 genes up- and down-regulated by FGF23 by microarray analysis.

| Gene Name | Symbol | *Fold change |
| --- | --- | --- |
| **UpRegulated Genes** |  |  |
| Early growth response 1 | EGR1 | +12.1 |
| Heme oxygenase (decycling) 1 | HMOX1 | +4.4 |
| Cytochrome P450, family 24, subfamily a, polypeptide 1 | CYP24A1 | +3.9 |
| Complement factor D (adipsin) | CFD | +2.8 |
| Uncoupling protein 1 | UCP1 | +2.6 |
| Heparin-binding EGF-like growth factor | HBEGF | +2.6 |
| Cytochrome P450, family 4, subfamily a, polypeptide 14 | CYP4A14 | +2.5 |
| FBJ osteosarcoma oncogene | FOS | +2.4 |
| Cytochrome c oxidase, subunit VIIIb | COX8B | +2.4 |
| Lipocalin 10 | LCN10 | +2.3 |
| Predicted gene | 9330164J24RIK | +2.2 |
| Polo-like kinase 3 | PLK3 | +2.2 |
| Zinc finger protein 36 | ZFP36 | +2.1 |
| Cell death-inducing DNA fragmentation factor, alpha subunit-like effector A | CIDEA | +2.1 |
| Predicted gene | A630005A06RIK | +2.0 |
| Ring finger protein 186 | RNF186 | +2.0 |
| Chemokine (C-X-C motif) ligand 1 | CXCL1 | +2.0 |
| Predicted gene | LOC382363 | +1.9 |
| RIKEN cDNA 1700034O15 gene | 1700034O15RIK | +1.9 |
| Regulator of calcineurin 1 | RCAN1 | +1.9 |
| **DownRegulated Genes** |  |  |
| Immunoglobulin kappa constant | IGK-C | -4.5 |
| Predicted gene | LOC100047628 | -3.6 |
| Predicted gene | IGH-6 | -3.5 |
| RIKEN cDNA 1810063B05 gene | 1810063B05RIK | -2.7 |
| Kinesin family member 20B | KIF20B | -2.5 |
| Zinc finger protein 810 | ZFP810 | -2.5 |
| Major facilitator superfamily domain containing 2 | MFSD2 | -2.5 |
| Hexosaminidase B | HEXB | -2.3 |
| Camello-like 3 | CML3 | -2.3 |
| Similar to Ig kappa chain V-V region K2 precursor | LOC636944 | -2.2 |
| Predicted gene | EG633640 | -2.2 |
| Protein phosphatase 1, catalytic subunit, beta isoform | PPP1CB | -2.2 |
| Olfactomedin 4 | OLFM4 | -2.2 |
| Transmembrane emp24 domain trafficking protein 2 | TMED2 | -2.2 |
| Expressed sequence | AI747699 | -2.2 |
| Similar to Ig kappa chain V-VI region NQ2-6.1 | LOC636752 | -2.1 |
| Cytochrome P450, family 4, subfamily a, polypeptide 12a | CYP4A12A | -2.1 |
| Predicted gene | LOC193533 | -2.1 |
| Predicted gene | LOC329575 | -2.1 |

*Fold change represents change in gene expression in microarray datasets in FGF23 treated 1-hr sample when compared to vehicle-treated sample.
