# Supplementary material for: Characterization of FGF23-Dependent Egr-1 Cistrome in the Mouse Renal Proximal Tubule
Source: PLoS One. 2015 Nov 20;10(11):e0142924. doi: 10.1371/journal.pone.0142924 (PMC4654537; doi:10.1371/journal.pone.0142924)
Supplement: S4 Table — (DOCX) [file pone.0142924.s005.docx]

**S4 Table.** List of top 20 genes up- and down-regulated by FGF23 in ChIP-Seq and microarray datasets.

| Gene Name | Symbol | *Fold Change |
| --- | --- | --- |
| **UpRegulated Genes** | | |
| Early growth response 1 | EGR1 | +12.1 |
| Heme oxygenase (decycling) 1 | HMOX1 | +4.4 |
| Cytochrome P450, family 24, subfamily a, polypeptide 1 | CYP24A1 | +3.9 |
| Heparin-binding EGF-like growth factor | HBEGF | +2.6 |
| FBJ osteosarcoma oncogene | FOS | +2.4 |
| Ring finger protein 186 | RNF186 | +2.0 |
| Predicted gene | 6820408C15RIK | +1.8 |
| Heat shock 70kDa protein 1A | HSPA1A | +1.7 |
| Protein Regulator of cytokinesis 1 | PRC1 | +1.6 |
| Predicted gene | 1700020N18RIK | +1.6 |
| parvin, beta | PARVB | +1.5 |
| delta-like 3 | DLL3 | +1.5 |
| natural killer cell granule protein 7 | NKG7 | +1.5 |
| Zinc finger protein 408 | ZFP408 | +1.4 |
| cyclin-dependent kinase inhibitor 1A | CDKN1A | +1.4 |
| activating transcription factor 3 | ATF3 | +1.4 |
| Spermine synthase | SMS | +1.4 |
| Predicted gene | 2210008F06RIK | +1.4 |
| Late cornified envelope 1M | LCE1M | +1.4 |
|  |  |  |
| **DownRegulated Genes** |  |  |
| Zinc finger protein 810 | ZFP810 | -2.5 |
| Beta-hexosaminidase subunit beta | HEXB | -2.3 |
| protein phosphatase 1, catalytic subunit, beta isozyme | PPP1CB | -2.2 |
| phosphoribosylaminoimidazole carboxylase | PAICS | -2.0 |
| acetoacetyl-CoA synthetase | AACS | -2.0 |
| Adenylate kinase isoenzyme 4 | AK3L1 | -2.0 |
| UDP-N-acteylglucosamine pyrophosphorylase 1 | UAP1 | -1.9 |
| Splicing factor, arginine/serine-rich 5 | SFRS5 | -1.9 |
| Cordon-bleu WH2 repeat protein-like 1 | COBLL1 | -1.9 |
| V-ral simian leukemia viral oncogene homolog A | RALA | -1.9 |
| Lon peptidase 2 | LONP2 | -1.9 |
| ADP-ribosylation factor (ARF)-like-6 | ARL6 | -1.9 |
| Diphosphoinositol polyphosphate phosphohydrolase 2 | NUDT4 | -1.9 |
| Predicted gene | 0610009B22RIK | -1.9 |
| Ubiquitin-conjugating enzyme E2Q family member 2 | UBE2Q2 | -1.9 |
| Nucleophosmin | NPM1 | -1.9 |
| Transmembrane 9 superfamily member 3 | TM9SF3 | -1.9 |
| Nucleoside diphosphate linked moiety X-type motif 12 | NUDT12 | -1.9 |
| Nucleoside diphosphate linked moiety X-type motif 19 | NUDT19 | -1.9 |

*Fold change represents change in gene expression in microarray datasets in FGF23 treated 1-hr sample when compared to vehicle-treated sample. Fold change in ChIP-seq dataset is not shown.
