# Supplementary material for: Characterization of FGF23-Dependent Egr-1 Cistrome in the Mouse Renal Proximal Tubule
Source: PLoS One. 2015 Nov 20;10(11):e0142924. doi: 10.1371/journal.pone.0142924 (PMC4654537; doi:10.1371/journal.pone.0142924)
Supplement: S5 Table — (DOCX) [file pone.0142924.s006.docx]

**S5 Table.** Ingenuity Pathway Analysis- Intersection of ChIP-seq and microarray datasets.

| **Top Networks** | | | | | |
| --- | --- | --- | --- | --- | --- |
| **#** | **Associated Network Function** | | **Score** | | |
| 1 | Hematological disease, organismal injury and abnormalities, cardiovascular system development an function | | 110 | | |
| 2 | Energy production, lipid metabolism, small molecule biochemistry | | 106 | | |
| 3 | Cellular assembly and organization, neurological disease, skeletal and muscular disorders | | 105 | | |
| 4 | Cell death and survival, post-translational modification, amino acid metabolism | | 90 | | |
| 5 | Gene expression, cellular growth and proliferation, post-translational modification | | 72 | | |
| **Top canonical pathways** | | | | | |
| **#** | **Pathway** | **P value** | | **Ratio** | |
| 1 | Molecular mechanisms of cancer | 2.37E-07 | | 53/354 (15%) | |
| 2 | Breast cancer regulation by stathmin 1 | 1.48E-05 | | 30/182 (16.5%) | |
| 3 | JAK/STAT signaling | 3.9E-05 | | 16/72 (22.2%) | |
| 4 | Integrin signaling | 7.16E-05 | | 29/188 (15.4%) | |
| 5 | NRF2-mediated oxidative stress | 9.46E-05 | | 26/163 (16%) | |
| **Top upstream regulators** | | | | | |
| **#** | **Regulator** | **P value** | | | **# molecules** |
| 1 | TP53 | 1.81E-07 | | | 134 |
| 2 | miR-340-5p | 3.05E-07 | | | 141 |
| 3 | Plicamycin | 8.00E-07 | | | 21 |
| 4 | HNF4a | 8.81E-07 | | | 182 |
| 5 | nocodazole | 3.14E-06 | | | 18 |
| **Top diseases and biological functions** | | | | | |
| **#** | **Diseases and disorders** | **P value** | | | **# molecules** |
| 1 | Cancer | 6.13E-08 - 5.75E-03 | | | 1027 |
| 2 | Neurological disease | 3.46E-07 - 5.13E-03 | | | 174 |
| 3 | Psychological disorders | 8.04E-07 - 3.66E-03 | | | 106 |
| 4 | Gastrointestinal disease | 1.14E-06 - 5.13E-03 | | | 741 |
| 5 | Skeletal and Muscular Disorders | 7.16E-06 - 5.13E-03 | | | 141 |
| **#** | **Molecular and Cellular Functions** | **P Value** | | | **# molecules** |
| 1 | Cell death and survival | 3.34E-10 - 6.26E-03 | | | 428 |
| 2 | Cellular growth and proliferation | 4.18E-10 - 6.22E-03 | | | 438 |
| 3 | DNA replication, recombination and repair | 4.88E-07 - 6.16E-03 | | | 114 |
| 4 | Cellular assembly and organization | 5.95E-07 - 5.86E-03 | | | 251 |
| 5 | Gene expression | 1.10E-06 - 6.22E-03 | | | 286 |
| **#** | **Physiological system development and function** | **P Value** | | | **# molecules** |
| 1 | Organismal survival | 4.86E-07 - 4.86E-07 | | | 290 |
| 2 | Connective tissue development and function | 9.25E-06 - 6.22E-03 | | | 139 |
| 3 | Tissue development | 1.26E-04 - 6.22E-03 | | | 252 |
| 4 | Hair and skin development and function | 1.77E-04 - 5.76E-03 | | | 68 |
| 5 | Embryonic development | 1.96E-04 - 6.26E-03 | | | 237 |
