# Supplementary material for: Characterization of FGF23-Dependent Egr-1 Cistrome in the Mouse Renal Proximal Tubule
Source: PLoS One. 2015 Nov 20;10(11):e0142924. doi: 10.1371/journal.pone.0142924 (PMC4654537; doi:10.1371/journal.pone.0142924)
Supplement: S6 Table — (DOCX) [file pone.0142924.s007.docx]

**S6 Table.** Ingenuity Pathway Analysis- Intersection of Chipseq, microarray and Col4a3^-/-^ mice datasets

| **Top Networks** | | | |
| --- | --- | --- | --- |
| **#** | **Associated Network Function** | | **Score** |
| 1 | Amino acid metabolism, Small molecule biochemistry & Cellular assembly and organization | | 113 |
| 2 | DNA replication, recombination and Repair & Energy metabolism | | 69 |
| 3 | Cell death and survival, cellular compromise and development | | 60 |
| 4 | Organismal development, cellular growth and proliferation & tissue development | | 49 |
| **Top canonical pathways** | | | |
| **#** | **Pathway** | **P value** | **Ratio** |
| 1 | Renal Cell Carcinoma Signaling | 9.59E-05 | 6/65 (9.2%) |
| 2 | NRF2-mediated oxidative stress response | 1.07E-04 | 9/163 (5.5%) |
| 3 | Jak/Stat signaling | 1.7E-04 | 6/72 (8.3%) |
| 4 | Stat3 pathway | 1.83E-04 | 6/73 (8.2%) |
| 5 | Mitochondrial dysfunction | 1.85E-04 | 26/163 (5.8%) |
| **Top upstream regulators** | | | |
| **#** | **Regulator** | **P value** | **# molecules** |
| 1 | INSR | 1.12E-07 | 56 |
| 2 | Mono-(2ethylhexyl)phtalate | 1.35E-06 | 24 |
| 3 | BAPTA-AM | 3.28E-06 | 44 |
| 4 | PPARGC1A | 3.49E-06 | 43 |
| 5 | PDGFBB | 4.26E-06 | 61 |
| **Top diseases and biological functions** | | | |
| **#** | **Diseases and disorders** | **P value** | **# molecules** |
| 1 | Developmental disorder | 3.00E-07-1.14E-02 | 36 |
| 2 | Hereditary disorder | 3.00E-07-1.14E-02 | 37 |
| 3 | Metabolic Disease | 3.00E-07-1.14E-02 | 33 |
| 4 | Organismal injury and abnormalities | 4.33E-07-1.14E-02 | 60 |
| 5 | Renal and urological disease | 4.33E-07-1.14E-02 | 18 |
| **#** | **Molecular and Cellular Functions** | **P Value** | **# molecules** |
| 1 | Cellular Assembly and organization | 1.01E-09-1.14E-02 | 42 |
| 2 | Cellular function and maintenance | 1.01E-09-1.13E-02 | 41 |
| 3 | Amino acid metabolism | 1.14E-08-1.14E-02 | 25 |
| 4 | Small molecule biochemistry | 1.14E-08-1.14E-02 | 67 |
| 5 | Lipid metabolism | 7.21E-07-1.14E-02 | 43 |
| **#** | **Physiological system development and function** | **P Value** | **# molecules** |
| 1 | Embryonic development | 5.62E-07-1.14E-02 | 36 |
| 2 | Organismal development | 5.62E-07-1.14E-02 | 54 |
| 3 | Digestive system development and function | 5.60E-06-1.14E-02 | 23 |
| 4 | Hepatic system development and function | 5.60E-06-1.14E-02 | 17 |
| 5 | Organ morphology | 5.60E-06-1.14E-02 | 31 |
